# Clinical presentations and management of COVID-19 infected children seen in a district health facility in Kambia, northern Sierra Leone

**DOI:** 10.11604/pamj.supp.2020.37.1.26312

**Published:** 2020-10-27

**Authors:** Hammed Hassan Adetola, David Ishola, Muhammed Olanrewaju Afolabi, Joseph Bangura, Isaac Gibril Sesay, Richmonda Pearce, Isaac Garrick, Bomposseh Kamara, Bailah Leigh, Brian Greenwood, Deborah Watson-Jones

**Affiliations:** 1 London School of Hygiene and Tropical Medicine/College of Medicine and Allied Health Sciences Research Collaboration, Kambia, Sierra Leone,; 2 Kambia District Health Medical Team, Ministry of Health and Sanitation, Freetown, Sierra Leone,; 3 Kambia Government Hospital, Kambia, Kambia District, Sierra Leone,; 4 College of Medicine and Allied Health Services (COMAHS), University of Sierra Leone, Freetown, Sierra Leone,; 5 Mwanza Intervention Trials Unit, National Institute for Medical Research, Mwanza, Tanzania

**Keywords:** COVID-19, children, Sierra Leone

## Abstract

Studies reporting the clinical presentations of COVID-19 in children in sub-Saharan Africa are few, especially from resource-constrained countries. This case series reports the demographic and clinical characteristics and laboratory findings of confirmed cases of COVID-19 in children seen at a district hospital in Sierra Leone. This is a report of nine COVID-19 paediatric cases managed at a secondary level hospital in Kambia District, Northern Sierra Leone. Each child was detected by contact tracing after an infected adult was identified by the COVID-19 response team. The clinical symptoms at presentation, clinical courses, and treatments instituted and patient outcomes are discussed in the context of the facilities available at a typical West African district hospital. Nine out of 30 individuals with confirmed COVID-19 infection who presented to the hospital from 24 April to 20 September 2020 and who were admitted to the isolation center of the hospital were in the paediatric age group. The mean age (SD) and median (IQR) of the children were 69.0 ± 51.7months and 84.0 (10.5, 108.0) months, respectively; five (55.6%) were males. The children were asymptomatic or only had mild illnesses and none required intranasal oxygen or ventilatory support. In the five symptomatic children, the most common symptoms were fever (40%) and cough (40%). All children had normal haemoglobin, platelet and white blood cell (WBC) count. Four children had a positive malaria test and were treated with a complete course of anti-malaria medications. No child received steroid or had specific anti-COVID-19 treatment. All children stayed in the isolation center for 14 days and were re-tested for COVID-19 two weeks after initial diagnosis. No complications have been reported in any of them since discharge. The proportion of children among COVID-19 infected cases seen in a rural community in Sierra Leone was 30%. Fever was the most common symptom and malaria was confirmed in 40% of the infected children. This has significant implication on the diagnosis of COVID-19 in malaria-endemic settings and on how best to manage children who present with fever during the COVID-19 pandemic.

## Introduction

Since the outbreak of a novel coronavirus, severe acute respiratory syndrome coronavirus 2 (SARS-CoV-2) in Wuhan, China in December 2019, there has been a rapid global spread of the virus and COVID-19 through human-to-human transmission [1]. As of 30^th^ September 2020, more than 33 million confirmed cases of COVID-19 and 1 million deaths have been reported globally [2], while about 1.5 million confirmed cases and 36,000 deaths have been reported from Africa [3]. In the USA, children accounted for 10.3% of all COVID-19 cases as of 17^th^ September 2020, with more than 94,000 children tested positive for the virus in the first two weeks of September alone, and 18 died during the course of that month [4]. Current reports have shown that children aged below 17 years have milder symptoms and are less likely than adults to be hospitalised because of COVID-19 infection [5]. Nevertheless, a multisystem inflammatory syndrome in children (MIS-C) associated with COVID-19 has been reported in United Kingdom and Africa [6], necessitating better understanding of the epidemiology and management of children with positive COVID-19 tests.

As of 22^nd^ September 2020, Sierra Leone, a country heavily affected during the 2014-2016 Ebola outbreak in West Africa [7], had reported 2,183 confirmed cases of COVID-19 and 72 deaths. During this period, Kambia District (one of the 16 districts in Sierra Leone) recorded 30 confirmed cases, nine of whom were in the paediatric age group. Limited data exist on the number of confirmed paediatric COVID-19 cases in Africa. Despite sustained community transmission in most African countries, the number of COVID -19 confirmed cases and deaths in children is very low. This might be due to inadequate testing capacity, poor health and mortality records and poor recording of health record indices [8]. Given gradual relaxation of public health measures including lockdown, and return of children to schools in many African countries, better understanding of the epidemiology and clinical features of COVID-19 in paediatric populations, especially in malaria endemic regions, is essential to aid early recognition and prompt case management. We describe in this report the clinical presentations and management of nine paediatric cases of COVID-19 infection admitted to the isolation center of a secondary health facility in a district hospital in Sierra Leone. These nine children were tested due to having been in contact with COVID-19 positive adults, in line with the Sierra Leone COVID-19 surveillance and contact-tracing programme [9]. None of the children presented primarily to the hospital on account of any illness prior to the testing and diagnosis of COVID-19 infection during screening.

## Methods

This is a report of COVID-19 positive children managed at a district hospital in Kambia District, northern Sierra Leone, West Africa. The diagnoses were made from the throat swabs collected from suspected COVID-19 cases in children who were close contacts of confirmed adults that were tested by reverse transcriptase-polymerase chain reaction test (RT-PCR) [10] at the Disease Prevention and Control Center, Freetown, capital of Sierra Leone. The results were made available within 48 hours of sample collection and individuals had to self-isolate while awaiting the results. The admission and subsequent management of the children were implemented according to the Sierra Leone guidelines for the management of COVID-19 positive subjects, irrespective of the ages of the subjects involved. All children were quarantined in the isolation center because of logistic issues arising from one or more of their caregivers' admission to the isolation center of the hospital at the same time. After being diagnosed as a COVID-19 case, each child had a routine blood examination including a full blood counts, human immunodeficiency virus screening test, random blood sugar estimation and a malaria rapid diagnostic (RDT) and blood film microscopy. Chest X-ray (CXR) was done only, if clinically indicated. Epidemiological, demographic, clinical, laboratory, radiological, and treatment data were retrieved from the hospital medical records by the first author (HHA). Descriptive analysis of these data was performed.

## Results

**Figure 1:**
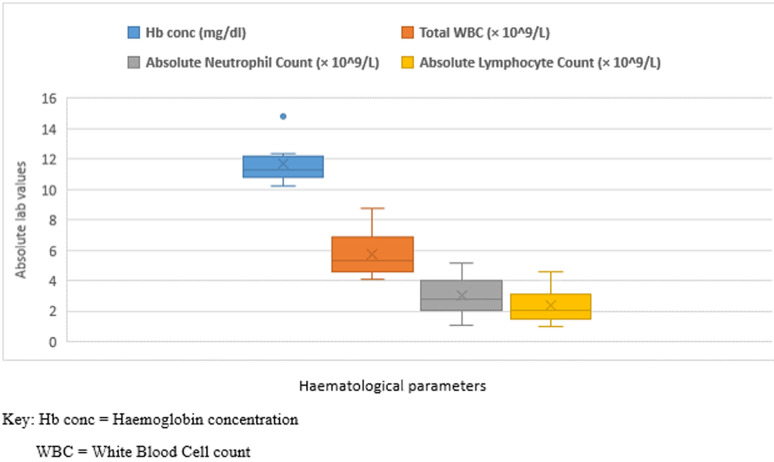
box-plots showing distribution of haematological parameters among nine confirmed paediatric COVID-19 cases on admission at health facility, Kambia, 2020

The demographic and clinical characteristics of the nine children are summarised in Table 1. The mean (SD) age of the children was 69.0 ± 51.7 months; median (IQR) age was 84.0 (10.5 - 108.0) months and their ages ranged from 7 days to 13 years. Five (55.6%) were male. None of the children had a history of traveling outside Sierra Leone, but all had contacts with a confirmed adult COVID-19 case. One case involved a first twin whose throat sample was collected within 24 hours of delivery to a confirmed COVID-19 positive mother, who eventually died from COVID-19 related complications. The second twin was negative for COVID-19. Another 2 months and 13 years old children had mothers who were positive for COVID-19. One child aged 7 years old had a neighbour who tested positive. All others had fathers who were diagnosed as COVID-19 positive. On admission, all but one child had a normal body temperature (36.5-37.5°C); this child who was aged 2 months, had mild fever of 37.9°C and swelling in the gluteal region secondary to an intramuscular injection (noticed a month earlier as an initial small swelling by the mother). Two children (aged 19 months and 8 years, respectively) presented with mild, dry, non-paroxysmal cough with no associated features of respiratory distress. The remaining five children had no symptoms on admission to the isolation center. In addition, the neonate was admitted into the isolation center for observation and management due to her age, peculiarity of susceptibility to infection and death of the mother. All children had a normal haemoglobin, platelet count and total white blood cell (WBC) count (Figure 1). However, one of the children (aged 8 years) had relative lymphocytopaenia and another (aged 13 years) had mild neutropaenia.

**Table 1:** demographic characteristics and clinical profile of nine paediatric COVID-19 cases, Kambia, 2020

SN	Age	Sex	RDT for Malaria	Symptoms on admission	Symptoms during admission
1	7 days	Female	Negative	Asymptomatic	Acute watery diarrhoea, fever, cough
2	2 months	Female	Negative	Fever, gluteal swelling	Gluteal abscess, cough, fever
3	19 months	Male	Negative	Cough	Sneezing, nasal discharge
4	4 years	Female	Positive	Asymptomatic	Asymptomatic
5	7 years	Male	Positive	Asymptomatic	Fever with rigor, vomiting, Nausea
6	8 years	Male	Positive	Cough	Headache, abdominal pain
7	8 years	Male	Negative	Asymptomatic	Malaise
8	10 years	Male	Negative	Asymptomatic	Fever, headache, Nausea
9	13 years	Female	Positive	Asymptomatic	Asymptomatic

Table 1 showed a slight male preponderance with ages ranging from 7 days to 13 years and six children (66.7%) had no clinical symptoms on admission

On admission, CXR was not done as no child had symptoms indicative of a chest infection. None of the patients required oxygen therapy or respiratory supports during admission. Four (44.4%) (aged 4, 7, 8 and 13 years, respectively) had positive results by malaria RDT on admission. They were subsequently treated with a complete course of artemether-lumefantrine. All children were HIV negative and all had a random blood sugar result within normal range. One child (7 days old) had an episode of acute watery diarrhea with moderate dehydration four days after admission and was managed with intravenous fluid replacement. Another child (aged 2 months) had a gluteal abscess (the same child with injection abscess noticed on admission but became an established abscess later) and was placed on antibiotics (suspension cefuroxime at 40 mg/kg per day in 2 divided doses). The child eventually had incision and drainage done, under full personal protective equipment (PPE) cover. Other children had non-specific mild symptoms which manifested 3 to 10 days after admission, as shown in Table 1. All the children were placed on routine haematinics and vitamin supplements. Hydroxyl-chloroquine, azithromycin, zinc or anti-retroviral drugs were not offered to any of the children. All nine children were re-tested for COVID-19 two weeks after the initial diagnosis and were PCR negative for COVID-19. All the children spent about 14 days stay in the isolation ward of the hospital before discharge. Follow-up of the children, has so far, shown no immediate post-COVID-19 complications.

## Discussion

This may be the first published report on the clinical profile and management of children with positive COVID-19 results in Sierra Leone. The children were tested because they were reported to have been exposed to close family members and/or other children with suspected or confirmed COVID-19. More boys were affected than girls and the ages of the children ranged from 7 days to 13 years. Only 40% of the children had any fever when first seen at the health facility, suggesting that undue emphasis should not be placed on temperature checks as a form of screening for COVID-19 [11]. Malaria was confirmed by a rapid diagnostic kit or microscopy in 40% of the children, although, only three out of the four children (with positive malaria test) presented with fever. One of the subjects had fever but was negative to malaria test, however, he was noted to have gluteal abscess, which may explain the fever. This has significant implication in malaria endemic settings, such as the study area, because malaria symptoms may overlap with COVID-19 symptoms, thereby leading to diagnostic dilemma [12]. Development of age-specific case definition and clinical management guideline is needed for the paediatric population. It is also pertinent to determine if malaria is a risk factor for COVID-19 in paediatric populations in Africa. If this is the case this would strengthen the need for effective malaria control programmes to support COVID-19 response efforts, and may help avoid a repeat of the 2014-16 West Africa Ebola outbreaks, where diversion of healthcare resources resulted in enhanced mortality from malaria [13]. This would strengthen continuous surveillance and control of COVID-19 infection among children in low-income countries. Also, the impact of malaria on the immune response to SARS-CoV-2, could predict whether the presence of malaria, and subsequent anti-malarial treatment, would impact on the effectiveness of a COVID-19 vaccine [14].

All the nine COVID-19 infected children had a mild clinical course. This supports several reports which suggest that, compared with adult patients, clinical manifestations of paediatric COVID-19 are less severe [15]. The reasons for the less severe nature of COVID-19 in children is not fully understood, however, exposure and host factors may play a role [15]. Recent evidence indicates that Angiotensin Converting Enzyme (ACE2) is the likely cell receptor for SARS-CoV-2 [16,17] and it is speculated that children are less sensitive to SARS-CoV-2, because the maturity and function (e.g. binding ability) of ACE2 in children may be lower than in adults. Children often experience respiratory infections (e.g. respiratory syncytial virus [RSV]) during wet seasons and it has been suggested that this may lead to higher levels of antibody against SARS-CoV-2 virus than in adults [18]. Furthermore, children´s immune systems are still developing and may respond to pathogens differently from adult immune systems [19]. Fortunately, none of the cases required administration of intranasal oxygen, steroids, intensive care unit admission or use of mechanical ventilator, as these are scarce resources in Sierra Leone, or needed administration of steroids. This study is limited by the small number of cases from a single center. However, similar findings have been observed in COVID-19 infected children in other parts of Sierra Leone (personal communications, Sierra Leone COVID-19 Emergency Response Team).

## Conclusion

This report has underscored the need for strengthening the ability of African health centers to manage moderate or severe paediatric cases of COVID-19, as more of such cases may be seen in the future.

### What is known about this topic

- Paediatric COVID-19 infection follows a mild course with occasional report of a multisystem inflammatory syndrome.

### What this study adds

- Awareness on the diagnostic dilemma and case management of children with confirmed SARS-CoV-2 and malaria infections in resource-limited settings.
